# Genome-wide *in vivo* screen identifies host molecule in promoting cancer metastasis

**DOI:** 10.1007/s13238-017-0391-9

**Published:** 2017-03-13

**Authors:** Yan Gu, Yanfang Liu, Xuetao Cao

**Affiliations:** 1 National Key Laboratory of Medical Immunology & Institute of Immunology, Second Military Medical University, Shanghai, 200433 China; 2 Department of Pathology, Changhai Hospital, Second Military Medical University, Shanghai, 200433 China; 3 Department of Immunology & Center for Immunotherapy, Institute of Basic Medical Sciences, Peking Union Medical College, Chinese Academy of Medical Sciences, Beijing, 100005 China

Metastasis, the movement of tumor cells from a primary site to progressively colonize distant organs, is the leading cause of cancer mortality. Emerging evidences show that tumor-educated host microenvironment cooperates with tumor cells during the multiple stage of metastasis, making tumor cells evade immune attack, resistant to apoptosis, and proliferate in distant organ (Liu et al., 2016; Quail and Joyce, 2013). This microenvironment consists of an elaborate array of inflammatory cells, fibroblastic cells, blood vessels, and the extracellular matrix (Joyce and Fearon, 2015; Liu and Cao, 2016). Re-educating the tumor-promoting microenvironment, especially activating the disabled immune system, is proved to be an effective strategy for treating cancer, such as immune checkpoint blockade (Pardoll, 2012; Tan et al., 2016). Therefore, uncovering the molecules of host microenvironment which may promote or inhibit tumor cell metastasis will be helpful to the design of cancer therapeutic approaches, although challenging but desperately needed.

In recent issue of Nature, Adams and colleagues performed a genome-wide *in vivo* screen of 810 mutant mouse lines to identify important metastatic regulators in host microenvironment (van der Weyden et al., 2017). This study was based on the “Sanger Mouse Genetics Project” in their institute to study new roles for a broad range of genes by generating more than 900 knockout mice (White et al., 2013). Therefore, the researchers used an “experimental metastasis assay” by intravenous administration of mouse metastatic melanoma cells in 810 mouse lines therein and then assessed the pulmonary metastasis. Compared with the wild-type mouse, they found that 15 mutant mouse lines showed significantly decreased pulmonary metastatic foci and 8 mutant mouse lines increased. Notably, most of these 23 genes were immune-related, indicating a key role of the immune system in microenvironmental regulation of metastasis.

They further focused on sphingosine-1-phosphate (S1P) transporter spinster homologue 2 (Spns2), as Spns2 mutant mice showed the most significant decrease in pulmonary metastasis. Spns2 is the cell-surface transporter of S1P, which regulates the egress of immune cells (T and B cells) from the lymphoid organs into the lymphatic vessels (Matloubian et al., 2004). Consistently, Spns2^tm1a/tm1a^ mice showed decreased S1P level in the serum, and a significant reduction in circulating T and B cells, but not other cell lineages. In the lung of mutant mice, T and B cells were also reduced, while NK cells were increased. Mice with lymphatic endothelial cell (LEC)-specific deletion of Spns2 (Spns2^tm1c/tm1c^; Lyve1^cre/+^ mice) had the similar phenotype with Spns2^tm1a/tm1a^ mice, indicating deficiency in LEC Spns2 was responsible for the reduction of peripheral T and B cells.

To determine how the alternated lymphocyte distribution could affect metastasis, intensive analysis of T cell subgroup was conducted. Interestingly, despite of their reduction in T cell numbers, Spns2^tm1a/tm1a^ mice showed an extremely higher percentage of anti-tumoral effector T cells. And increased production of interferon-γ (IFN-γ) and enhanced B16-F10 target cell killing was also found in CD8^+^ T cells from Spns2^tm1a/tm1a^ mice, suggesting a role of CD8^+^ T cells in the suppression of metastasis. However, by *in vivo* antibody depletion of CD8^+^ T cells, the number of metastatic foci in mutant mice was still less than that in wild-type mice. In consideration of elevated NK cells in the lung, researchers eliminated NK cells in these mice, which could not restore the pulmonary metastasis yet. Only when the mutant mice received depletion of both CD8^+^ T cells and NK cells, the number of metastatic foci showed no difference with the wild-type mice. Therefore, both CD8^+^ T cells and NK cells contributed to the reduced pulmonary metastasis in Spns2^tm1a/tm1a^ mice.

**Figure 1:**
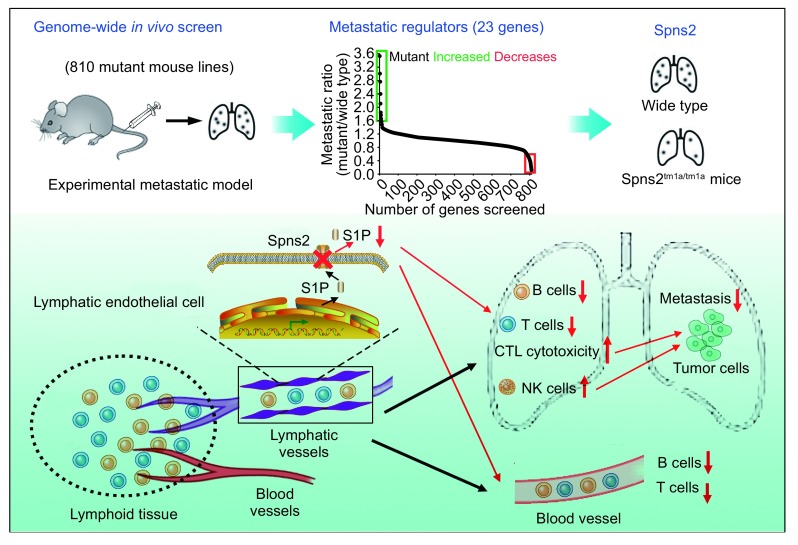
Genome-wide *in vivo* screen identifies host Spns2 in promoting cancer metastasis. Adams and colleagues identify 23 novel genes in host microenvironment to regulate metastasis by genome-wide *in vivo* screen using an experimental metastasis assay. Host Spns2 is demonstrated as an important metastatic promoter via modulating lymphocyte trafficking. CTL, cytotoxic T lymphocyte; NK cell, natural killer cell; S1P, sphingosine-1-phosphate; Spns2, S1P transporter spinster homologue 2

Taking together, this impressive study by Adams and colleagues identified several previously unknown molecules in host microenvironment to regulate metastasis through the powerful tool of genome-wide *in vivo* screen. They found host Spns2 plays a role in promoting metastasis via modulating lymphocyte trafficking (Fig. 1), outlining Spns2 as a promising therapeutic target against tumor metastasis, which may take the place of existing S1P pathway interventions to overcome their broad side-effect.

Immunotherapy is now proved to be quite effective in the treatment of metastatic cancer. Polarizing the activation of immune response in host microenvironment against tumor is promising (Chen et al., 2014). Thus, this work based on genome-wide *in vivo* screen provides a comprehensive view of the interaction between tumors and immune system and the regulation of the complex immune networks. In the future, more systematic and lager scale of host genes are needed to be included in this kind of *in vivo* screen. Also, the expression profiling of the metastatic prone organs may be combined with this *in vivo* screen approach to find the potential molecules involved in metastatic organisms. As to the translation of Spns2-targeting therapy into clinical application, there are still some questions to be further addressed: How is the expression and what is the function of Spns2 in other host cells? Do the alternated B cells and CD4^+^ T cells modulate metastasis in Spns2-deficient mice? How does Spns2 regulate NK cell distribution? In sum, microenvironmental regulation of tumor progression needs to be extensively investigated, which may provide novel therapeutic strategies to control metastasis.
